# *PHACTR1* Is a Genetic Susceptibility Locus for Fibromuscular Dysplasia Supporting Its Complex Genetic Pattern of Inheritance

**DOI:** 10.1371/journal.pgen.1006367

**Published:** 2016-10-28

**Authors:** Soto Romuald Kiando, Nathan R. Tucker, Luis-Jaime Castro-Vega, Alexander Katz, Valentina D’Escamard, Cyrielle Tréard, Daniel Fraher, Juliette Albuisson, Daniella Kadian-Dodov, Zi Ye, Erin Austin, Min-Lee Yang, Kristina Hunker, Cristina Barlassina, Daniele Cusi, Pilar Galan, Jean-Philippe Empana, Xavier Jouven, Anne-Paule Gimenez-Roqueplo, Patrick Bruneval, Esther Soo Hyun Kim, Jeffrey W. Olin, Heather L. Gornik, Michel Azizi, Pierre-François Plouin, Patrick T. Ellinor, Iftikhar J. Kullo, David J. Milan, Santhi K. Ganesh, Pierre Boutouyrie, Jason C. Kovacic, Xavier Jeunemaitre, Nabila Bouatia-Naji

**Affiliations:** 1 INSERM, UMR970 Paris Cardiovascular Research Center (PARCC), Paris F-75015, FRANCE; 2 Paris-Descartes University, Sorbonne Paris Cité, Paris 75006, FRANCE; 3 Cardiovascular research Center, Massachusetts General Hospital, Charlestown, MA 02114, USA, Program in Medical and Population Genetics, The Broad Institute of Harvard and MIT, Cambridge, MA 02142; 4 Department of Internal Medicine and Department of Human Genetics, University of Michigan, Ann Arbor, MI 48109, USA; 5 The Zena and Michael A. Wiener Cardiovascular Institute, Icahn School of Medicine, Marie-Josée and Henry R. Kravis Cardiovascular Health Center at Mount Sinai, One Gustave L. Levy Place, Box 1030 New York, NY 10029, New York, NY, USA; 6 Assistance Publique-Hôpitaux De Paris, Referral Center for Rare Vascular Diseases, Hôpital Européen Georges Pompidou, Paris, F-75015, FRANCE; 7 Assistance Publique-Hôpitaux De Paris, Department of Genetics, Hôpital Européen Georges Pompidou, Paris, F-75015, FRANCE; 8 Department of Cardiovascular Diseases, Mayo Clinic, Rochester, MN 55905, USA; 9 Dept. of Health Sciences, Genomic and Bioinformatics Unit, Viale Ortles 22/4, Milano, Chair and Graduate School of Nephrology, University of Milano, Division of Nephrology, San Paolo Hospital, Milano, 20142,ITALY; 10 Institute of Biomedical Technologies, Italian National Centre of Research, Via F.lli Cervi 93, 20090 Segrate - Milano; 11 Nutritional Epidemiology Research Group, Sorbonne-Paris-Cité, UMR University of Paris 13/Inserm U-557/INRA U-1125/CNAM, Bobigny, France F-93017, Bobigny, FRANCE; 12 Assistance Publique-Hôpitaux De Paris, Department of Cardiology, Hôpital Européen Georges Pompidou, Paris, F-75015, FRANCE; 13 Department of Cardiovascular Medicine, Cleveland Clinic Heart and Vascular Institute, Cleveland, OH 44195, USA; 14 Assistance Publique-Hôpitaux De Paris, Department of Hypertension, Hôpital Européen Georges Pompidou, Paris, F-75015, FRANCE; 15 INSERM, Clinical Investigation Center CIC1418, Hôpital Européen Georges Pompidou, Paris, F-75015, FRANCE; 16 Assistance Publique-Hôpitaux De Paris, Department of Pharmacology, Hôpital Européen Georges Pompidou, Paris, F-75015, FRANCE; University of Ottawa Heart Institute, CANADA

## Abstract

Fibromuscular dysplasia (FMD) is a nonatherosclerotic vascular disease leading to stenosis, dissection and aneurysm affecting mainly the renal and cerebrovascular arteries. FMD is often an underdiagnosed cause of hypertension and stroke, has higher prevalence in females (~80%) but its pathophysiology is unclear. We analyzed ~26K common variants (MAF>0.05) generated by exome-chip arrays in 249 FMD patients and 689 controls. We replicated 13 loci (P<10^−4^) in 402 cases and 2,537 controls and confirmed an association between FMD and a variant in the phosphatase and actin regulator 1 gene (*PHACTR1*). Three additional case control cohorts including 512 cases and 669 replicated this result and overall reached the genomic level of significance (OR = 1.39, P = 7.4×10^−10^, 1,154 cases and 3,895 controls). The top variant, rs9349379, is intronic to *PHACTR1*, a risk locus for coronary artery disease, migraine, and cervical artery dissection. The analyses of geometrical parameters of carotids from ~2,500 healthy volunteers indicate higher intima media thickness (*P* = 1.97×10^−4^) and wall to lumen ratio (*P* = 0.002) in rs9349379-A carriers, suggesting indices of carotid hypertrophy previously described in carotids of FMD patients. Immunohistochemistry detected PHACTR1 in endothelium and smooth muscle cells of FMD and normal human carotids. The expression of *PHACTR1* by genotypes in primary human fibroblasts showed higher expression in rs9349379-A carriers (N = 86, *P* = 0.003). *Phactr1* knockdown in zebrafish resulted in dilated vessels indicating subtle impaired vascular development.

We report the first susceptibility locus for FMD and provide evidence for a complex genetic pattern of inheritance and indices of shared pathophysiology between FMD and other cardiovascular and neurovascular diseases.

## Author Summary

Fibromuscular Dysplasia (FMD) is a vascular disease characterized by a succession of occlusions and dilatation of medium-sized arteries (e.g renal, carotid or coronary arteries) with important health consequences, mainly resistant hypertension and stroke. FMD is an atypical vascular disease because it is not associated with overweight or dyslipidemia and 80% of patients are early middle aged women. Our genetic study conducted in >1100 patients and >3800 controls demonstrate that a common variant rs9349379 located on chromosome 6 in the phosphatase and actin regulator 1 gene (*PHACTR1*) increases by ~40% the risk of FMD. This is the first time a genetic risk factor is reported for FMD because it has been longtime considered rare and potentially under a Mendelian mode of inheritance. We also show that rs9349379 correlates with the expression of *PHACTR1* in fibroblasts from FMD patients and controls. Interestingly, the same allele that increases the risk of FMD is at risk for cervical artery dissection and migraine, often reported in FMD patients but protective from myocardial infarction and coronary disease, where atherosclerosis is more common. The clear role of PHACTR1 in maintaining vascular well integrity is not fully elucidated. Using a specific antibody we detected PHACTR1 both on endothelial and smooth muscle cells of human FMD and control carotids, which suggests that PHACTR1 may have multiple functions depending on the cell type and the degree of atherosclerosis of the arteries.

## Introduction

Fibromuscular dysplasia (FMD) is a non-atherosclerotic and non-inflammatory vascular disease leading to stenosis, aneurysm, dissection, and/or occlusion of medium-sized arteries, in particular the renal and extracranial cerebrovascular arteries [1–3]. FMD predisposes to hypertension, transient ischemic attack and stroke [2,3]. Intriguingly, 75% to 90% of FMD patients are women[3,4] and FMD is increasingly considered to be a silent and under-diagnosed condition [5].

The angiography-based classification of renal FMD distinguishes between patients with multifocal stenoses, including the "string-of-beads” FMD pattern, and unifocal (or focal) FMD with corresponding differences in sex ratio, median age of diagnosis and smoking status [6,7].

The pathogenesis of FMD is unknown and there are strong arguments in favor of a genetic origin. We have previously reported familiality of FMD in ~10% of patients [8]. The US FMD registry has also described a family history in first or second-degree relatives for FMD (7%) and aneurysm (23%) [3]. Nonetheless, the unknown status of most of family members compromised heritability estimates and made the assessment of the genetic mode of inheritance difficult. Recently, we investigated the coding genomes from 16 affected siblings and excluded the existence of a major mutated gene in familial FMD [9]. The complexity of the diagnosis based on computed tomography angiography (CTA) and/or magnetic resonance angiography (MRA) and a general lack of awareness among affected patients and clinicians result in an under-diagnosis bias.

To advance our understanding of the etiology of FMD, we performed a genetic association study that identifies a first genetic susceptibility locus for FMD. In addition to the high prevalence of asymptomatic FMD (~3–6%) [3,10] and the existence of environmental modifiers (e.g female hormones, lifetime mechanical stress) our study provides genetic and functional evidence supporting for the first time a complex genetic basis for FMD.

## Results

### Association of rs9349379 in *PHACTR1* with FMD

We carried-out a multi-stage genetic association study, including a discovery and four validation case control cohorts, to identify genetic determinants of FMD. All FMD patients and controls were of European ancestry and have similar overall clinical features (Table 1). First, we analyzed 25,606 common (minor allele frequency ≥ 0.05) genetic variants in 249 FMD cases and 689 controls. Despite the small sample size, we also performed stratified analyses including only females (193 patients and 416 controls). Associations across chromosomes are summarized in Manhattan Plots (S2 Fig). In the global analysis, no SNP achieved the adjusted significance threshold for multiple testing (*P* = 1.95×10^−6^, S2 Fig). Nevertheless, the strongest association signal located on chromosome 6 (rs9349379, effect allele frequency (EAF) in cases = 0.70, odds ratio (OR) = 1.65, *P* = 1.47×10^−5^) surpasses the adjusted threshold in the female only analysis (OR = 1.99, *P* = 8.16 × 10^−7^, S2 Fig, S1 Table).

**Table 1:** Clinical features of the study populations.

Cohorts	N	Females (%)	Age at exam (yrs)	Multifocals (%)	HTN (%)	HTN diagnosis (yrs)	Study Design
RVDRC Cases	249	193 (78%)	44.12 ± 14.31	164 (66%)	228 (92%)	36.6 ± 13.65	Clinical recruitment
SU.VI.MAX controls	689	416 (60%)	49.80 ± 6.20	NR	NA	NA	Population based
ARCADIA cases	402	319 (79%)	42.46 ± 15.68	273 (68%)	341 (85%)	34.9 ± 15.15	Clinical recruitment
PPS3 controls	2,537	1012 (40%)	58.73 ± 5.94	NR	0	NR	Population based
Mayo Cases	143	119 (83)%	61.48 ± 13.43	NA	108 (76%)	58.75 ± 10.31	Clinical recruitment
Mayo Controls	333	286 (86%)	65.78 ± 10.50	NR	169 (51%)	62.52 ± 10.25	Clinical recruitment
UM/Cleveland Cases	304	295 (97%)	53.93 ± 10.45	254 (84%)	193 (63%)	NA	Clinical recruitment
UM/Cleveland Controls	289	280 (97%)	55.13 ± 9.97	NR	NA	NA	Clinical recruitment
DEFINE-FMD Cases	65	65 (100%)	57.03 ± 10.13	65 (100%)	41 (63%)	42.68 ± 13.05	Clinical recruitment
DEFINE-FMD Controls	47	47 (100%)	50.70 ± 9.38	NR	4 (9%)	55.25 ± 13.05	Clinical recruitment

HTN, Hypertension

NR, Not Relevant

NA, Not Available.

In addition to rs9349379, we selected for follow-up loci that showed suggestive association with FMD (*P<*10^−3^) and were located in or near either biological candidate genes (e.g extracellular matrix degradation) or previous cardiovascular genome-wide association (GWAS) signals. We also prioritized four SNPs located within one megabase interval around rs9349379, the top associated variant. The first follow-up study included 402 FMD patients from the ARCADIA registry, who had similar clinical characteristics to the patients of the discovery stage and were compared to 2,537 controls from PPS3 (Table 1). Of the 16 SNPs selected, 13 passed genotyping QC criteria in cases and controls. Three SNPs, all located in the *PHACTR1* locus, showed replicated association with FMD (rs9349379-A, *P* = 7.21 × 10^−4^, rs9369640-C, *P* = 8.45 × 10^−4^ and rs1332844-C, *P* = 1.72 × 10^−3^, S1 Table). Conditioned regression analyses for rs9349379 in the case control cohort including the discovery and first follow-up study samples indicated association signal redundancy for rs9369640 (*P* = 0.21) and rs1332844 (P = 0.24) with rs9349379 being the most statistically significant. Next, we investigated the association of rs9349379 with FMD in three additional and independent case-control cohorts from the USA. Overall, of the four follow-up studies, three showed a significant effect of rs9349379 on the risk of FMD; all studies showed consistent direction of effect of the FMD risk allele rs9349379-A being more prevalent in FMD cases (Table 2). The association meta-analysis included 1,154 FMD patients and 3,895 controls and indicated an overall OR of 1.39 for rs9349379 (EAF = 0.69 in global cases sample), with a high level of significance (*P* = 7.36×10^−10^) that is below the genomic threshold and no indices for heterogeneity between studies (*P* = 0.574, Table 2).

**Table 2:** Association of rs9349379 in *PHACTR1* with FMD in five independent case-control cohorts.

							Additive model		Recessive model	
Study	Cohorts	*n*	AA	AG	GG	EAF (A)	OR (95% CI)^a^	P Value^a^	OR (95% CI)^a^	P Value^a^
Discovery	RVDRC Cases	249	124	103	22	0.70	1.65 (1.32–2.07)	1.47 × 10^−5^	1.87 (1.39–2.52)	3.39 × 10^−5^
	SU.VI.MAX controls	689	237	341	111	0.59				
Follow-up	ARCADIA	393	183	170	40	0.68	1.32 (1.12–1.54)	7.21 × 10^−4^	1.38 (1.11–1.71)	0.003
	PPS3 controls	2537	982	1174	381	0.62				
	Mayo Cases	143	62	65	16	0.66	1.37 (1.02–1.83)	0.034	1.94 (0.91–4.16)	0.09
	Mayo Controls	333	116	159	58	0.59				
	UM/Cleveland Cases	304	145	130	29	0.69	1.31 (1.03–166)	0.026	1.43 (0.96–2.14)	0.08
	UM/Cleveland Controls	289	116	135	38	0.63				
	DEFINE-FMD Cases	65	41	18	6	0.77	1.39 (0.77–2.49)	0.276	1.38 (0.99–1.91)	0.05
	DEFINE-FMD Controls	47	22	22	3	0.70				
Meta-analysis^b^	All Cases	1154	555	486	113	0.69	1.39 (1.25–1.54)	7.36 × 10^−10^	1.50 (1.3–1.73)	1.39 × 10^−8^
	All Controls	3895	1473	1831	591	0.61				

OR, Odds Ratio

CI, Confidence Interval

EAF, Effect Allele Frequency.

^a^Odds Ratio (OR) and P values were computed by logistic regression under the additive and recessive genetic model.

^b^Meta-analysis was performed using inverse variance-weighted method. Heterogeneity between cohorts was tested using Cochran’s Q statistics and was not significant (P_add_ = 0.574;P_rec_ = 0.483).

As for rare variants (MAF < 5%), we observed 62,767 polymorphic variants in the discovery case control analyses that we analyzed using the SKAT-O gene-based association test. None of the 9,967 genes covered with at least 2 polymorphic variants showed significant association with FMD after Bonferroni correction (P = 5.02 × 10^−6^, strongest gene P = 1.42 × 10^−4^), or biological candidacy among best scoring genes, which discourages follow-up genotyping or sequencing efforts in larger samples.

### Association of rs9349379 with carotid parameters in PPS3

**Fig 1:**
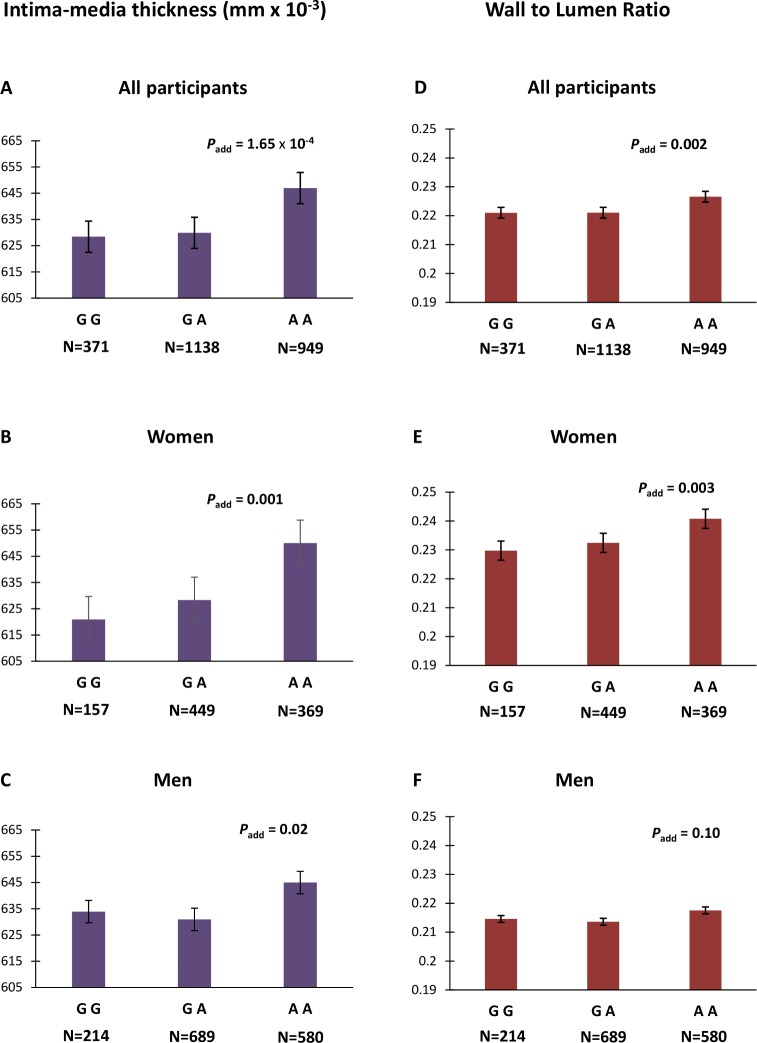
Association between rs9349379 and artery thickness in healthy controls. Intima-media thickness and wall to lumen ratio are presented by genotype in all (A,D) women (B,E) and men (C,F) participants. We indicated p-values for the linear regression analyses under the additive model adjusted for age, sex and body surface area.

Here we aimed to characterize the association of rs9349379 with artery thickness and stiffness using a non-invasive method in 2,458 healthy volunteers. Genetic association analyses indicate significant association between the FMD increasing risk allele rs9349379-A and greater intima-media thickness (IMT) (β_add_ = 11.65 μm, *P*_*add*_ = 1.65×10^−4^, Fig 1) and wall to lumen ratio (WLR) (β_add_ = 0.004, *P*_*add*_ = 0.002, Fig 1), and decreased circumferential wall stress at DBP (β_add_ = -0.72, *P*_*add*_ = 0.004, S2 Table). Interestingly, despite their smaller number (N = 975) compared to males (N = 1,483), females present more accentuated effect of the rs9349379-A allele on IMT (β_add_ = 14.83 μm, *P*_*add*_ = 0.001) and WLR (β_add_ = 0.006, *P*_*add*_ = 0.003). However, we detected no significant interaction with sex. rs9349379-A also associated with increased wall cross-sectional area (β_add_ = 0.24 mm^2^, *P*_*add*_ = 8.67×10^−4^, S2 Table), peripheral SBP (β_add_ = 0.70 mmHg, P_add_ = 0.009, S2 Table) and central pulse pressure (β_add_ = 0.62 mmHg, *P*_*add*_ = 0.002, Fig 1).

### *PHACTR1* expression studies

**Fig 2:**
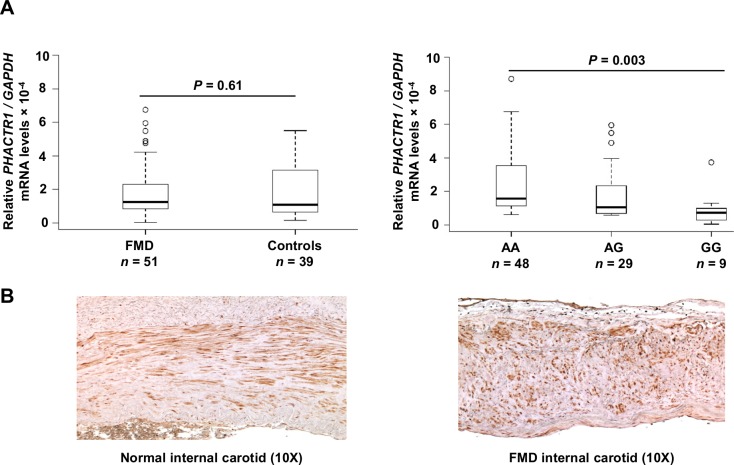
*PHACTR1* mRNA expression and immunostaining. (A) *PHACTR1* mRNA expression in human fibroblasts. *PHACTR1* mRNA levels were determined by RT-qPCR in cultured fibroblasts from controls (N = 39) and FMD cases (N = 51). *GAPDH* expression was used as control for normalization and data are expressed as mean fold change of *PHACTR1* relative to *GAPDH*. There is a non-significant trend toward overexpression of *PHACTR1* in FMD cases compared to controls (*P* = 0.61, Mann-Whitney test) whereas significant differences are uncovered after stratification by genotypes (*P* = 0.003, Kruskal-Wallis test). (B) Immunostaining of normal and FMD internal carotid using anti-PHACTR1 antibody. PHACTR1 was detected in endothelium and smooth muscle cells in the media. Staining is mostly cytoplasmic with regular alignment in normal carotids and typical disorganized cellular structure in media of FMD carotid.

Variant rs9349379 resides in the fourth intron of the phosphatase actin regulator 1 gene (*PHACTR1*) in a putative regulatory sequence. We assessed *PHACTR1* expression using quantitative real time PCR in mRNAs from primary cultured human fibroblasts. *PHACTR1* expression did not differ in fibroblasts from FMD patients compared to age and sex matched controls (Fig 2). However, stratifying by rs9349379 genotype indicated increased expression in individuals carrying the FMD risk allele rs9349379-A (*P*_*add*_ = 0.003, Fig 2). At the protein level, using a specific antibody, we found that PHACTR1 is expressed in endothelial and medial smooth muscle cells of carotid arteries from both normal and FMD patients (Fig 2).

### Functional role of *Phactr1* in vascular development

**Fig 3:**
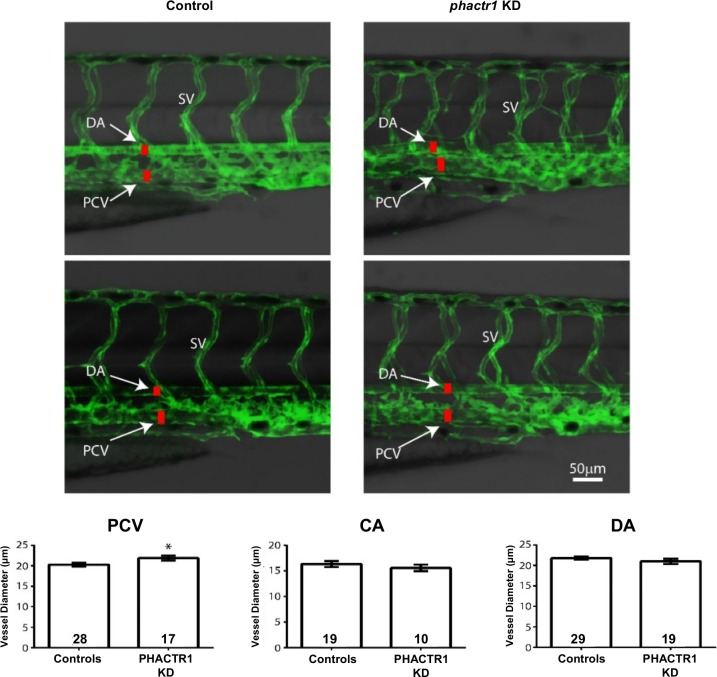
*phactr1* modulation in zebrafish affects vascular dimensions and patterning. Two-dimensional projections obtained from z-series confocal images in the trunk of control and phactr1 knockdown (KD) zebrafish embryos (two representative images per condition). Green represents the vascular endothelium as marked by EGFP. Greyscale represents the corresponding DIC bright field image of the fish trunk. DA: Dorsal aorta, SV: Segmental vessel, PCV: Posterior cardinal vein. Quantification of inner vascular diameter for the dorsal aorta (DA), posterior cardinal vein (PCV) and caudal artery (CA). (*) represents *P*<0.05.

Given the unclear role for *PHACTR1* in vascular development and maintenance, we assessed the effect of *PHACTR1* perturbation in the zebrafish. Morpholino injected embryos had undetectable *phactr1* transcript levels at 72 hours post fertilization while overall morphology was unchanged (S3 Fig). Compared to control injected zebrafish, *phactr1* knockdown resulted in a marked disorganization of the developing hepatic portal vein and the segmental vessels in the developing trunk (Fig 3 and S3 Fig). Further analysis of the diameter of three major peripheral vessels, the dorsal aorta, caudal artery and posterior cardinal vein, demonstrated a nearly 8% dilatation (*P*<0.05) of the posterior cardinal vein in *phactr1* suppressed embryos.

## Discussion

Our study describes for the first time the genetic association of rs9349379, a common variant in *PHACTR1*, with arterial fibromuscular dysplasia (FMD). We demonstrate that this common variant increases by ~40% the risk of FMD in five independent case-control studies. These findings are based on genetic data from 1,154 FMD patients and 3,895 controls, the largest investigation conducted so far to elucidate the genetic basis of this intriguing vascular disease with unknown etiological origin and challenging clinical features.

To date, the genetic investigation on FMD was limited to the screening of candidate genes involved in rare vascular and arterial syndromes, and/or the study of underpowered series of patients.[9,11,12] For long considered a rare disease, FMD has also been hypothesized to be under the genetic control of highly penetrant genetic defects. Our study provides the first evidence for a complex genetic pattern of inheritance for FMD, involving a common genetic allele (frequency = 0.60 in the general population). The identification of a genetic susceptibility locus for FMD supports the concept that this disease is controlled by a large number of genetic determinants in strong interaction with various environmental factors, including female sex and mechanical stresses.

We provide genetic evidence for increased IMT, narrowed artery lumen and no change in arterial stiffness, even after adjusting for blood pressure, in healthy volunteers carrying the rs9349379-A, the FMD risk allele. These genetic associations support the observation of carotid concentric hypertrophy in FMD risk carriers, especially females. This finding is consistent with previous arterial features reported in FMD patients when compared to age, sex and SBP matched controls.[13] Circumferential wall stress was decreased in rs9349379-A carriers, showing that hypertrophy may overcome the moderate increase in blood pressure. Of note, the effect size of the FMD risk allele on IMT corresponds to approximately five years of aging for the IMT, when reported to sex and age reference values for arterial geometry,[14] suggesting potentially an accelerated arterial aging in carriers of the rs9349379-A FMD risk allele.

*PHACTR1* was previously identified by several genome-wide association studies (GWAS) as a risk locus for cardiovascular and neurovascular diseases. *PHACTR1* is a confirmed susceptibility locus for coronary artery disease (CAD) and myocardial infarction (MI),[15,16] migraine[17,18] and more recently cervical artery dissection (CeAD), a rare condition defined as a mural hematoma in a carotid or vertebral artery and a cause of stroke.[19] Of note, the association of rs9349379 is in the opposite direction for FMD, CeAD and migraine, with rs9349379-A at risk, when compared to CAD and MI with rs9349379[G] allele at risk. Migraine and CeAD share several clinical features with FMD. Migraine is also more prevalent in females than in males and it is reported by a third of FMD patients.[3] CeAD is an important risk factor for subarachnoid hemorrhage stroke, as is cerebrovascular FMD.[3] The investigation of a large series of CeAD patients indicated that cervical FMD is reported in 5.6% of the patients[20] and carotid dissection is the presenting manifestation in 12.1% of patients of the US FMD registry.[3] In contrast to migraine and CeAD, the implication of the same common variant in *PHACTR1* in CAD/ MI and FMD is rather unexpected, though it involved a different allele at the same genetic variant. The clinical link between FMD and CAD/MI is less obvious, except for the high proportion of FMD in rare forms of MI involving spontaneous coronary artery dissection, that also present in young females without atherosclerotic risk factors.[21–23] By definition FMD does not involve atherosclerotic stenosis that is concomitant with CAD and MI through pathogenesis processes (e.g dyslipidemia and inflammation). Future comprehensive genetic investigation by full GWAS for FMD will allow the assessment of the putative consistency and opposition of effects between risk loci for these cardiovascular and neurovascular diseases.

The rs9349379 is intronic to *PHACTR1* in a putative noncoding regulatory sequence. *PHACTR1* encodes a phosphatase and actin regulator protein and its function is not fully elucidated. Here we describe significant correlation between rs9349379 genotypes and *PHACTR1* expression in human fibroblasts from FMD patients and controls. This association is consistent with the expression quantitative trait loci (eQTL) data from several artery beds of the Genotype-Tissue Expression (GTEx) project and a recent study where increased expression is reported in human coronary arteries from donors carrying the rs9349379-A.[16] Using a genome-editing technique with CRISPR-Cas9 applied to human umbilical vein endothelial cells, the deletion of the sequence containing rs9349379 caused a 35% decrease in *PHACTR1* expression and impaired the fixation of the myocyte enhancer factor-2 (MEF2), supporting a functional regulatory effect of this genetic variant *in vitro*.[16] However, other transcription factors may also bind to this site, as the knockdown of the expression of two members of the MEF2 family did not change *PHACTR1* expression.[24] We note that ENCODE ChIP-Seq data derived from genomes extracted from two immortalized cell lines after stimulation by estradiol indicates several consistent peaks for ESR1 suggesting a putative regulation of PHACTR1 by this female hormone. This putative regulation that needs to be established experimentally is consistent with the high proportion (~75–90%) of women among FMD patients.

Molecular studies have also linked PHACTR1 to cell adhesion and migration in angiogenesis via vascular endothelial growth factor (VEGF) stimulation.[25,26] In contrast to other studies,[26,27] Beaudoin et al reported a lack of evidence for the induction of PHACTR1 expression in endothelial cells with pro-angiogenic (VEGF), pro-inflammatory stimulations or shear stress.[16] Our immunohistochemistry staining of PHACTR1 indicates the presence of PHACTR1 both in endothelial and smooth muscle cells of normal and FMD carotid arteries. The genetic implication of *PHACTR1* in FMD, the subtle impairment in the development of vasculature in zebrafish, in addition to the evidence from recent GWAS describing an increasing number of loci with genes involved in vessel wall biology in CAD and MI[28] all support PHACTR1 plays a key etiological role in vascular structure. Recent work on *PHACTR1* regulation in atherosclerosis showed strong expression in human atherosclerotic plaque macrophages lipid-laden foam cells, adventitial lymphocytes and endothelial cells.[29] This group also describes the absence of PHACTR1 in SMCs of healthy and athesclerotic aortas, which is not supported by our immunostaining on medium-sized arteries using a different anti-body, though they have detected the expression of an intermediate transcript in SMCs. Further investigation is required to understand this discrepancy that might reflect *PHACTR1* is much more abundantly expressed in macrophages from atherosclerotic plaques, where this population of cells is highly represented and biologically active, which is different from the FMD and healthy vessels without atherosclerosis. Thus, according to rs9349379 genotype, the manifestation of one specific vascular disease depends on multiple and particular environmental triggers (e.g. female specific hormonal context, mechanical movements of medium size arteries in the case of FMD) that are still to be specifically determined for each of these cardiovascular and neurovascular diseases.

In summary, our study provides genetic and functional evidence to support *PHACTR1* as a first susceptibility locus for FMD. Further functional exploration of this locus and more comprehensive genetic investigation through genome-wide association will provide additional predisposing loci to FMD and help understanding the etiological mechanisms of non-atherosclerotic arterial stenosis.

## Methods

### Ethics Statement

We obtained individual written informed consent from all participants included in France and the USA case control studies.

French FMD patients (RVDRC and ARCADIA) from the European Hospital Georges Pompidou (HEGP) are part of the ARCADIA/PROFILE protocol that was approved by the Ile-De-France research ethics committee (Comité de Protection des Personnes: CPP d’île de France) on 03/04/2009 (ID: 2009-A00288-49). The ethics committee of the Paris-Cochin hospital approved the protocols of the SU.VI.MAX study (CCPPRB No. 706) and PPS3 (CPP No. 2007-A01386-47). PPS3 is declared at ClinicalTrials.gov (Identifier: NCT00741728). Mayo Clinic case control study is part of the Vascular Diseases Biorepository study and was approved by the Mayo I Mayo Clinic Institutional Review Boards (IRB # 08–008355). UM/Cleveland case control study was approved by each institution IRB protocols: the University of Michigan IRB number HUM00044507 and the Cleveland Clinic IRB number 10–318. The DEFINE-FMD study was approved by the Human Research Ethics Committee of the Icahn School of Medicine at Mount Sinai (Study ID: HS#13-00575/GCO#13–1118 and is registered with ClinicalTrials.gov Identifier: NCT01967511.

### Patients and control populations

We used a three-stage association design. First, we performed an exome-chip based genetic association in 249 French FMD patients (RVDRC cohort) and 689 controls from SU.VI.MAX [30]. Second, we followed-up 13 loci in an independent set of 402 French patients (ARCADIA registry) and 2,537 controls from PPS3 [31]. Three additional studies from the USA totaling 512 patients and 669 controls were used for further replication: Mayo Clinic cohort [32], University of Michigan (UM)/Cleveland Clinic cohort and the DEFINE-FMD study. All participants are of European ancestry and presented similar clinical characteristics (Table 1) and homogeneous diagnosis, exclusion and inclusion criteria.

#### RVDRC and ARCADIA cases

We analyzed unrelated FMD followed-up at the Rare Vascular Diseases Reference Center (RVDRC) of the European Hospital Georges Pompidou (HEGP), Paris, France. We ascertained patients from the ARCADIA (Assessment of Renal and Cervical Artery DysplasIA) register, an ongoing national FMD registry at the HEGP, Paris. The diagnosis of FMD in RVDRC and ARCADIA patients was established using clinical information from the medical history, the interpretation of angiography and/or computed tomography scan of arterial beds after the exclusion of other causes of arterial stenosis such as atherosclerosis, Takayasu disease and Elhers Danlos syndrome and neurofibromatosis type 1. Given the complexity of the interpretation of imaging of vascular diseases, a local panel of experts including clinicians from the departments of hypertension, radiology, vascular medicine and medical genetics validated the diagnosis of FMD.

#### SU.VI.MAX and PPS3 controls

Controls in discovery stage are French Europeans from the SU.VI.MAX study as previously described [30]. PPS3 controls were 2,537 participants ascertained from the Paris Prospective Study 3 (PPS3), an ongoing observational French prospective study evaluating the possible implication of numerous vascular health parameters in cardiovascular disease in healthy subjects aged from 50 to 75 years undergoing a standard health examination in a large preventive medical center that is subsidized by the French national insurance system for salaried workers [31].

#### Mayo Case-Control study

Potential FMD cases were identified from the Mayo vascular disease bio-repository. Electronic health records of 501 patients were manually reviewed by clinicians and the diagnosis was confirmed for 191 patients according to criteria in [1], of whom 159 had DNA available and 143 (83% females) had high-density genotyping data. Patients were matched for sex and age (+/- 2 years) to 333 controls without known FMD or other atherosclerotic vascular diseases.

#### UM/Cleveland Clinic case control study

Patients were recruited at the University of Michigan (UM) and/or the Cleveland Clinic. UM FMD cases were recruited into an IRB-approved study through a referral clinic at the University of Michigan and through self-referral to the study. The Cleveland Clinic cases were enrolled among consecutive patients seen at a dedicated FMD referral clinic. Clinical diagnosis of FMD was ascertained by a vascular medicine specialist after review of diagnostic imaging and prior to blood sample collection. DNAs from healthy controls without vascular disease were obtained from the Cleveland Clinic GeneBank, which was approved by the Cleveland Clinic IRB. Genomic DNA was isolated from a peripheral blood sample and analyzed as described[33].

#### The DEFINE-FMD Study

Eligible cases were females with an imaging-confirmed diagnosis of multifocal FMD and who fulfilled other accepted diagnostic criteria [1]. Healthy controls were matched to FMD cases according to age and sex, required to be receiving ≤ 2 blood pressure medications, have a body mass index < 35kg/m2 and to be non-smokers. Healthy controls underwent physical exam and those with bruits; unexplained hypertension or other cardiovascular findings were excluded. Exclusion criteria for all subjects included male gender, unifocal FMD, use of immunosuppressive agents, major comorbidities, diseases that may confound genetic/genomic analyses (i.e. Crohn’s disease, multiple sclerosis etc.) or any other form of heritable vascular disease (i.e. Ehlers-Danlos, Marfan, Loeys-Dietz).

### Genotyping

Genotypes were generated using the Illumina-HumanExome-12v1 array in RVDRC, SU.VI.MAX and PPS3,[34] Illumina-Human-Omni-Express-Exome in the DEFINE-FMD Study participants, Illumina-Infinium-Human-CoreExome in Mayo Clinic cohorts and by individual genotyping in ARCADIA (KASP technology) and UM/Cleveland Clinic cohorts (Taqman). We applied quality control (QC) filters to the discovery cases and controls as recommended[35] (individual’s call rate < 97%, extreme heterozygosity, sex-discordant, duplicate or relatedness unsing PLINK (version 1.07).[36] Individuals with non-European ancestry were detected and excluded (54 cases and 9 controls) using the EIGENSTRAT program[37] and visualized by principal components analysis (PCA) including HapMap phase 3 samples. Cases and controls displayed a comparable distribution after ancestry QC (S1 Fig). From 240,748 successfully genotyped SNPs, we excluded monomorphic variants (n = 129,890), call rate < 99%, deviation from Hardy-Weinberg Equilibrium in cases and/or controls (*P* < 10^−5^) and minor allele frequency (MAF)<0.05 in controls. The final analysis included 25,606 variants (25,138 autosomal and 468 X-linked) in a sample of 249 FMD cases and 689 controls. Comparable individuals and markers QC was applied to follow-up cohorts using arrays (PPS3, Mayo Clinic case control cohorts, the DEFINE-FMD study) or individual genotyping (ARCADIA and UM/Cleveland cases control cohort).

### Carotid geometry and stiffness measurements in PPS3 cohort

Carotid parameters were measured and calculated as previously reported [34]. Briefly, a 10 MHz 128 transducer linear array probe was positioned on the carotid area. Measurements were performed on a 4 cm segment of the right common carotid artery, 1 cm proximal to the bifurcation/sinus throughout the cardiac cycle for 6 seconds. A longitudinal section showing clear interfaces for blood/intima and media/adventitia was obtained. The system allows real-time radiofrequency signal analysis with operator-independent determination of external diameter (Dext), internal diameter (Dint), and intima-media thickness (IMT) on 128 lines throughout the cardiac cycle. Distension was measured on 14 lines at high-pulsed radiofrequency (600 Hz). The axial resolution was 34 μm for diameter, 17 μm for IMT, and 1.7 μm for distension.[38] Aortic blood pressure was estimated from the distension waveform according to van Bortel et al.[39] The distensibility coefficient, representing the elastic properties of the artery as a hollow structure, was calculated as dLCSA/(LCSA×central PP), where LCSA is the lumen cross-sectional area and central PP is the aortic pulse pressure. Carotid stiffness (Cstif) was calculated as DC^−0.5^ and circumferential wall stress as diastolic blood pressure×Dint/2×IMT. WCSA is wall cross-sectional area. Young's elastic modulus is calculated as DC^−1^x3(1+LCSA/WCSA). Here we examined the association of one genetic variant (rs9349379) with 14 interdependent traits in a sub-sample of 2458 participants (975 females and 1483 males). Hypertensive participants (BP over 140 and/or 90 mmHg, and/or use of antihypertensive treatments) were excluded to avoid confounding with genetic effects.

### Statistical analyses

We tested the association with FMD using logistic regression under the additive genetic model as implemented in PLINK[36] (version 1.07) in discovery and follow-up studies. In the discovery analysis, we included the first five principal components axes as co-variates to control for hidden population stratification. The Bonferroni adjusted threshold for significance was set to *P* = 1.95×10^−6^ to account for multiple testing of 25,606 common variants. We used the inverse variance-weighted method for meta-analysis implemented in Metal [40]. Gene-based analyses of rare variants was performed using SKAT-O method as described in.[41] Heterogeneity was assessed with Cochran's Q statistics. We tested the association of rs9349379 with carotid parameters in 2,458 normotensive subjects (975 females and 1483 males) from PPS3 using a linear regression on an additive genetic model including age, sex, body surface area (BSA), and mean blood pressure (MBP) as covariates when relevant. Before performing the analyses, all parameters (all quantitative traits) were quantile-transformed to a standard normal distribution. The 14 traits fall into three main carotid parameters categories: geometry (intima-media-thickness, external and internal carotid diameters, wall to lumen ratio and circumferential wall stress), arterial stiffness (stiffness, cross-sectional distensibility, Young’s elastic modulus, cross-sectional compliance, and wall cross-sectional area) and central blood pressure (systolic, diastolic and aortic blood pressure). Considering that carotid parameters are interdependent, we applied a Bonferroni correction (*P*_*adj*_ = 0.017) for the three main categories as recommended [34].

### *PHACTR1* expression in fibroblasts

We analyzed the expression of PHACTR1 from fibroblasts of skin biopsy samples of 104 individuals (51 FMD, 39 controls, 12 undetermined). Genotypes and detectable expression levels were available in 86 individuals.

Fibroblasts of the DEFINE-FMD Study were derived from skin biopsy samples using standard explant techniques from 103 individuals (46 FMD and 57 controls). Briefly, skin biopsy samples were dissected into small pieces and cultured under cover slips in DMEM/F12 media containing 20% fetal bovine serum, 1% antibiotic-antimycotic solution, 1% 200 mM L-glutamine, 1% 100mM Sodium pyruvate, and 1% MEM Non-Essential Amino Acids (all from Life Technologies, Grand Island, NY, USA) at 37°C in 5% CO_2_. Cell culture medium was replaced every 48 hours and confluent cells from passages 2–3 were used for RNA extractions. RNA was then converted to cDNA using iscript cDNA synthesis kit (Bio Rad, Hercules, CA, USA) and quantitative real time PCR (qRT-PCR) was performed using SYBR green fastmix low rox (Quanta Biosciences, Gaithersburg, MD, USA). Fibroblasts from the University of Michigan were obtained through the Coriell Biorespository (Camden, NJ, USA) for 20 subjects with FMD and apparently 20 healthy control subjects and matched for passage number. DNAs and genotypes were not available. Confluent cells from passages 6–8 were used to extract RNA and generate cDNAs to assess *PHACTR1* expression according to culture and extraction protocol described for the DEFINE-FMD study. Fibroblasts from the Department of genetic, HGEP study were derived from skin biopsy samples from patients followed up for unknown arterial diseases where FMD and Elhers Danlos syndrome were discarded. FMD status is not available. SNP rs9349379 was genotyped by direct forward and reverse sequencing (BigDye Terminator kit v3.1 cycle sequencing kit) and run on an ABI Prism 3730XL DNA Analyzer Sequencer (Perkin Elmer Applied Biosystems, Foster City, CA). Cell culture and RNA extraction conditions and reagent were identical to the DEFINE-FMD study. Informed consent was obtained from all patients of all centers.

In total, we analyzed the expression of *PHACTR1* from fibroblasts of skin biopsy samples of 104 individuals (51 FMD, 39 controls, 12 undetermined), and 86 individuals had genotypes and relevant expression levels. The expression of PHACTR1 transcript variant 1 (NCBI Reference Sequence: NM_030948.2) and *GAPDH*, which was used as normalization control, was determined by RT-qPCR following the MIQE guidelines.[42] All expression studies from the three laboratories used the same following primers: PHACTR1 fwd ATGACCGCAGGGCAGATAAG, rev TTCGGATGGCAGCTTTGTCT; GAPDH fwd GGGTGTGAACCATGAGAAGTATGA, rev GGTGCAGGAGGCATTGCT. The efficacy and efficiency of amplifications for PHACTR1 and GAPDH were equivalent as determined by the linearity tests: (*PHACTR1* efficacy 1.97 and efficiency 98.7%); (GAPDH efficacy 2.04 and efficiency 102%). The specificity of amplification products was verified by the presence of a single peak in the melting temperature curve analysis. Outliers displaying *GAPDH* Cq values different from the mean or a ΔCq value >14 (Cq value PHACTR1 –Cq value *GAPDH*) were filtered out. Good correlations for RT and technical qPCR replicates were obtained. Given that data obtained from three independent laboratories was equivalent: mean Cq for *GAPDH* 17,41 +/- 0,65 and mean Cq for *PHACTR1* was 30.46 +/- 1.19, we pooled them as indicated to gain power, especially for the correlation by genotypes where few samples per study were GG carriers. Data is presented as the mean fold change in the expression of PHACTR1 relative to GAPDH, calculated using the formula 2^-ΔCq^, as recommended.[43] Statistical analyses were performed using a non-parametric Mann-Whitney test for single comparison between FMD cases and controls and Kruskal Wallis test for comparisons according to rs9349379 genotypes.

### PHACTR1 Immunostaining

Protein staining for PHACTR1 was performed on paraffin embedded artery samples using primary anti-PHACTR1 antibody (Sigma-Aldrich, St Louis, MI, USA) and revealed using an ABC peroxidase kit with diaminobenzidine (Vector laboratories, Burlingame, CA, USA). The paraffin blocks of arterial tissues were obtained from surgical pathology archives of the HEGP as remnants of the regular diagnostic procedure (2 renal arteries, 1 internal carotid, 1 popliteal and 1 femoral artery). FMD arteries belonged to patients who had surgical reparation for aneurysm resection (1 carotid, 1 radial, 1 coeliac, 1 ulnar and 1 pancreaticoduodenal artery). The arterial tissues were fixed in formalin and embedded in paraffin. Antigen retrieval were performed by incubating tissue sections in alkaline solution (Dako, Trappes, France) for 40 minutes at 94°C in a hot water bath. The sections were then incubated for 60 minutes with the primary anti-PHACTR1 rabbit polyclonal antibody diluted at 1/50. For revelation we used ABC peroxidase kit with diaminobenzidine (Vector laboratories, Burlingame, CA, USA).

### Zebrafish experiments

Anti-*phactr1* morpholino was designed to target the exon7/intron7 boundary of zebrafish *phactr1* transcript. A non-targeting morpholino of equivalent length but differing nucleotide composition was injected at equivalent concentrations as a control. For injections, 0.8nL of control of anti-*phactr1* morpholino was injected into single cell embryos of the Tubingen/AB strain. For confirmation of morpholino efficacy, primers spanning the targeted boundary were used to amplify cDNA constructed from the isolated RNA of injected 72 hour post fertilization embryos. All the morphological analyses as well as the knockout status of the fish were performed blinded to the experimenter. For imaging studies, fish were reared under standard conditions with addition of phenylthiourea to media in order to inhibit pigmentation. At 72hpf, high-speed videography was performed as described[44] used to determine the diameter of major vessels. Measurement of vessels was manually performed using ImageJ. For confocal reconstructions of the embryonic vasculature, embryos obtained from lines expressing EGFP driven by the *flk* promoter were used. Confocal images were obtained on a Nikon A1SiR confocal. Two-dimensional projections of maximal intensity (head and torso) or averaged intensity (trunk) were used to visualize the overall vascular architecture using ImageJ.

## Supporting Information

S1 Fig Principal component analysis using genotypes from FMD cases and controls of the discovery stage. Data used is post quality control filtration for non-European ancestry origin and relatedness
(TIFF)

S2 Fig Association between Single-Nucleotide Polymorphisms (SNPs) and Fibromuscular Dysplasia (FMD). (A) Association between SNPs and FMD in global analysis. (B) Association between SNPs and FMD in females only. The red line indicates the Bonferroni adjusted significance threshold for 25,606 common variants tested set to *P* = 1.95 × 10^−6^.
(TIFF)

S3 Fig Phenotypic evaluation of *phactr1* knockdown zebrafish. (A) RT-PCR evaluation of splice alteration observed following microinjection of phactr1 morpholino (PHACTR1 KD). (B) Brightfield micrographs of overt morphology at 60 hours post fertilization. (C) Two-dimensional projections obtained from z-series confocal images in the head and trunk of control and phactr1 knockdown zebrafish embryos. Green represents the vascular endothelium as marked by EGFP. Greyscale represents the corresponding DIC brightfield image of the fish head and trunk region. HPV indicates the relative position of the developing hepatic portal vein
(TIFF)

S1 Table Association of SNPs selected for follow-up in ARCADIA and PPS3 case control analyses. SNP, Single Nucleotide polymorphism; Chr, Chromosome; EA, Effect allele; OR, Odds-Ratio; CI, Confidence Intervalle. ^a^For genic SNPs, the relevant gene is listed; for intergenic SNPs nearest upstream and downstream genes are listed. ^b^SNPs added despite a P value in the discovery stage were above the threshold given their location in or near *PHACTR1*, the most associated locus.
(DOCX)

S2 Table Association of rs9349379 in *PHACTR1* with carotid artery parameters in PPS3 controls. ^a^All (N = 2,458), Female (N = 975) and Males only (N = 1,483) participants of the PPS3. β_q.t._ is the effect size estimated using quantile-transformed values and β is the effect size estimated using untransformed values. P values indicated are for the analyses of quantile transformed values. Age, sex, body surface area (BSA), smoking status and cholesterol were included as covariables in all regression models. MBP was an additional covariable for the following carotid geometry (IMT, Dext, Dint and WLR) and arterial stiffness (Stiffness, Dist, Comp and WCSA) parameters.
(DOCX)
