# Supplementary figures and images for: PHACTR1 Is a Genetic Susceptibility Locus for Fibromuscular Dysplasia Supporting Its Complex Genetic Pattern of Inheritance
Source: PLoS Genet. 2016 Oct 28;12(10):e1006367. doi: 10.1371/journal.pgen.1006367 (PMC5085032; doi:10.1371/journal.pgen.1006367)

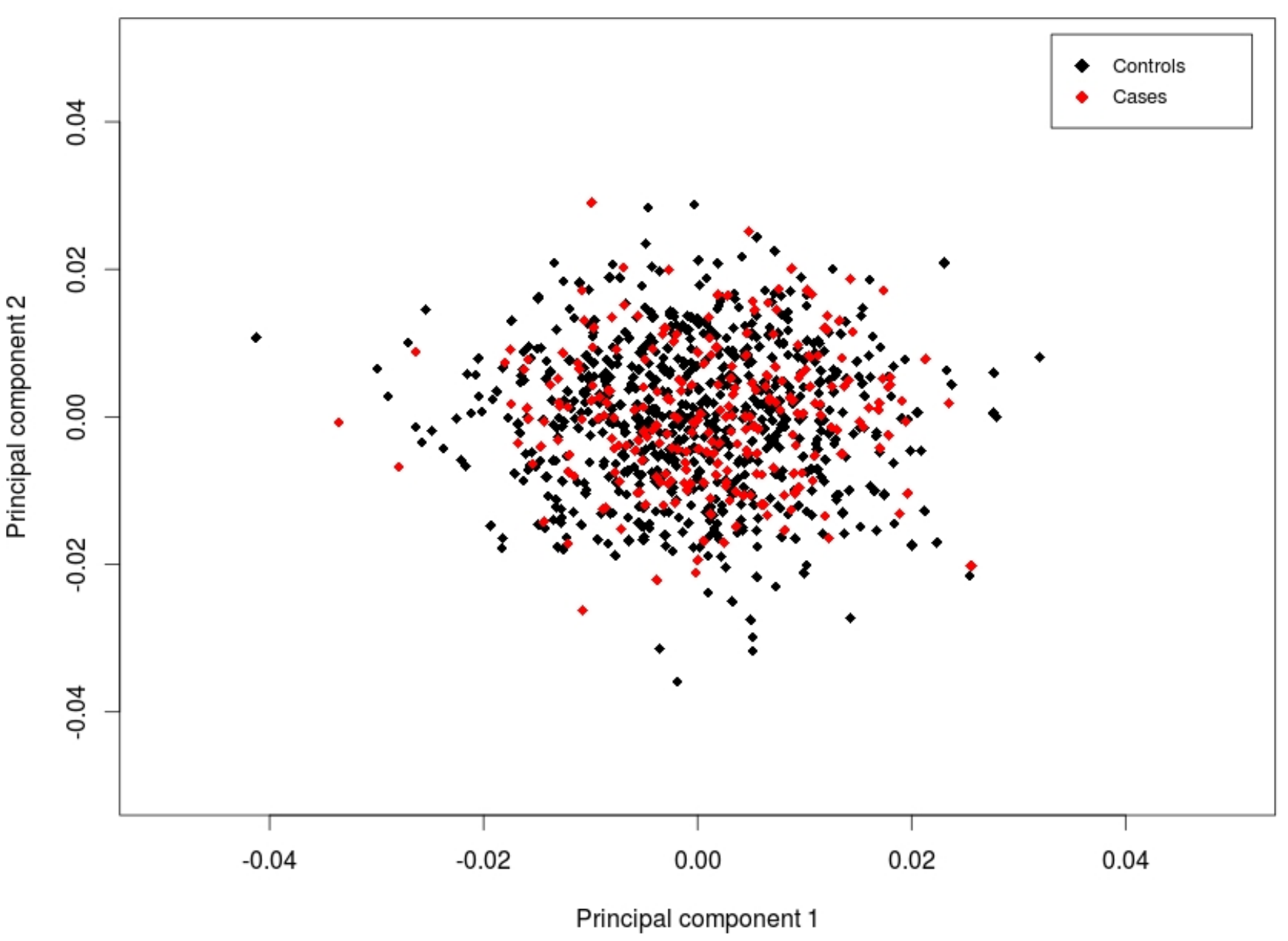

Supplement: S1 Fig — Data used is post quality control filtration for non-European ancestry origin and relatedness (TIFF) [file pgen.1006367.s001.tiff]

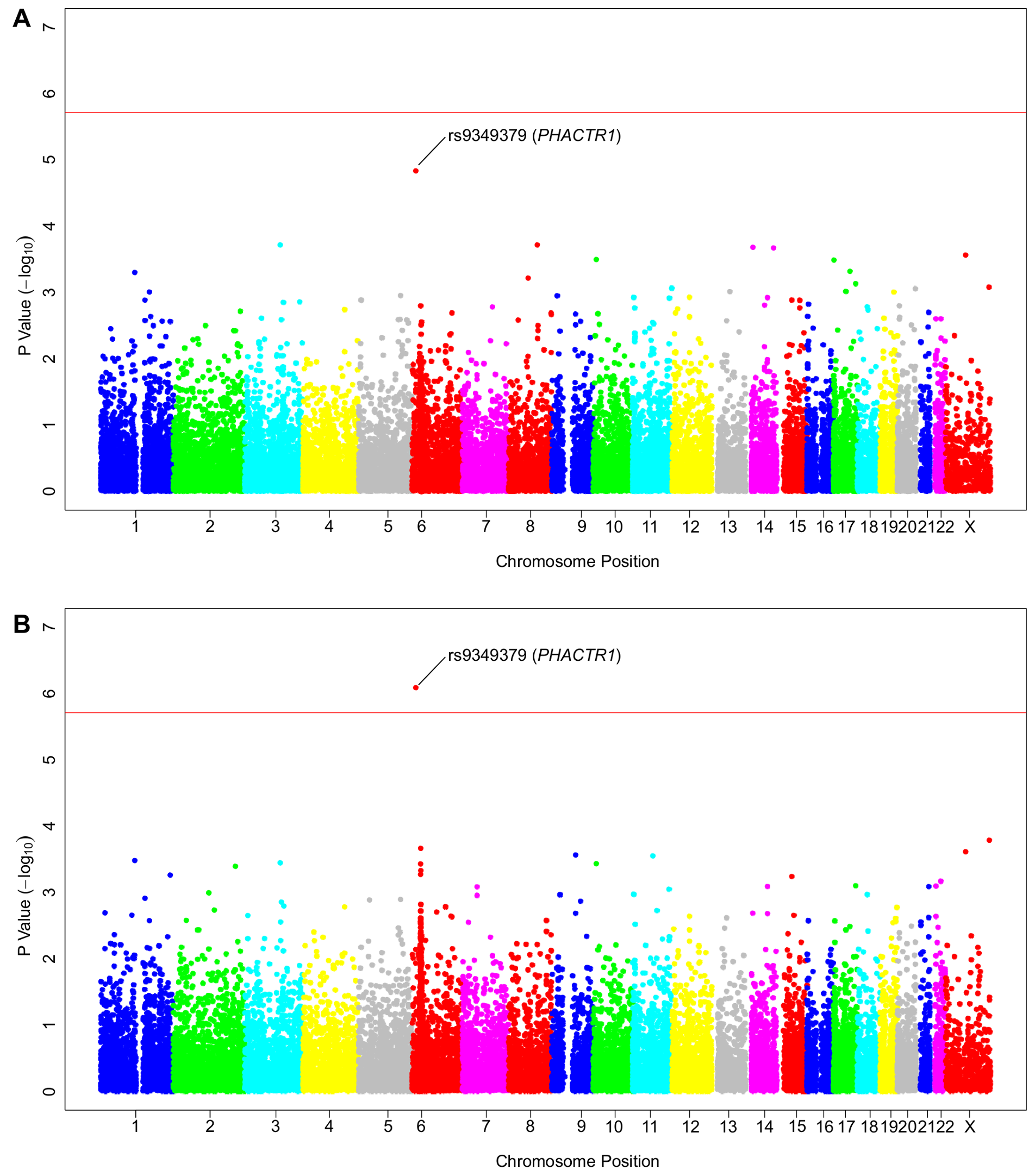

Supplement: S2 Fig — (A) Association between SNPs and FMD in global analysis. (B) Association between SNPs and FMD in females only. The red line indicates the Bonferroni adjusted significance threshold for 25,606 common variants tested set to P = 1.95 × 10^−6^. (TIFF) [file pgen.1006367.s002.tiff]

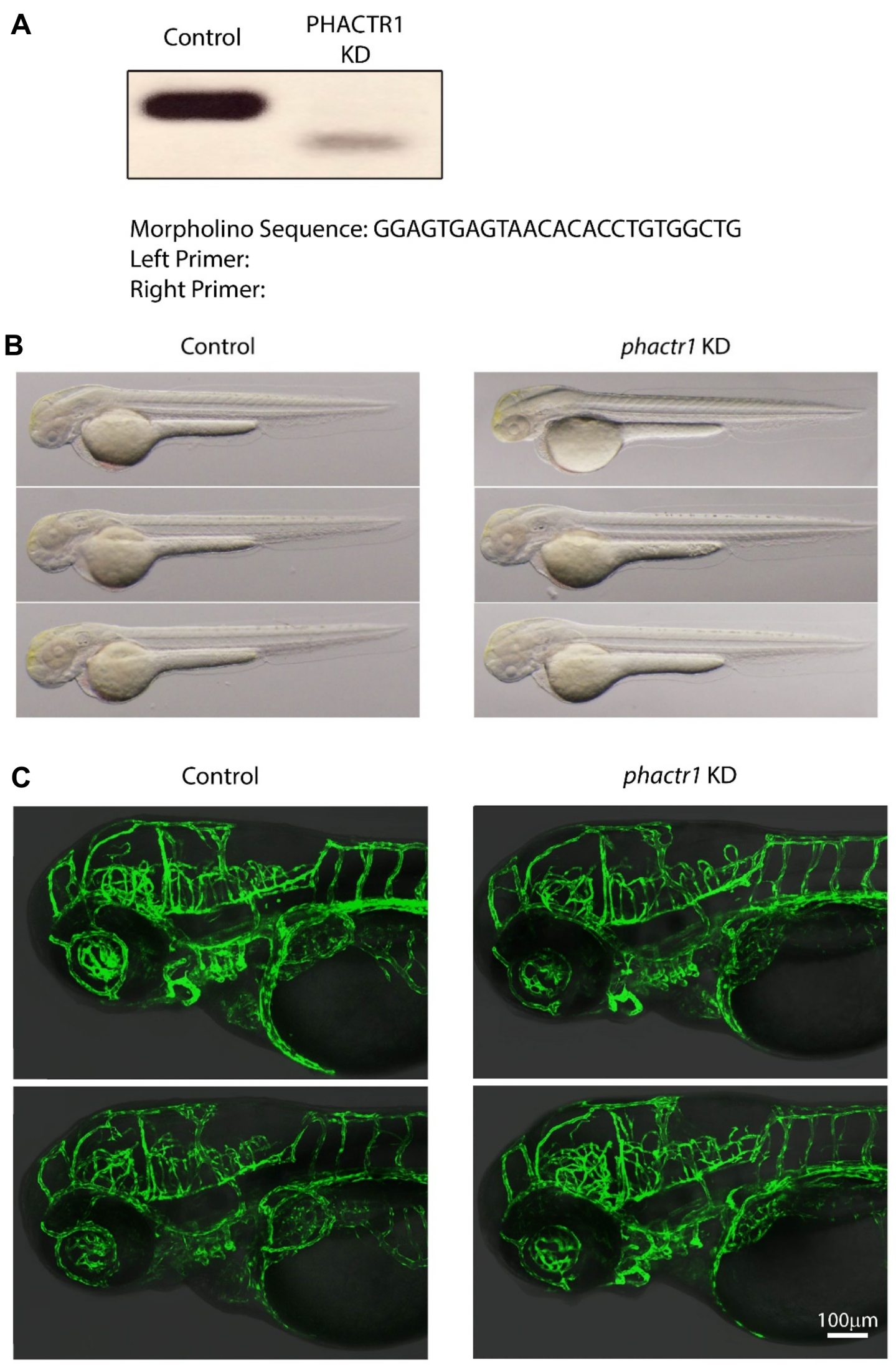

Supplement: S3 Fig — (A) RT-PCR evaluation of splice alteration observed following microinjection of phactr1 morpholino (PHACTR1 KD). (B) Brightfield micrographs of overt morphology at 60 hours post fertilization. (C) Two-dimensional projections obtained from z-series confocal images in the head and trunk of control and phactr1 knockdown zebrafish embryos. Green represents the vascular endothelium as marked by EGFP. Greyscale represents the corresponding DIC brightfield image of the fish head and trunk region. HPV indicates the relative position of the developing hepatic portal vein (TIFF) [file pgen.1006367.s003.tiff]
