# Supplementary material for: PHACTR1 Is a Genetic Susceptibility Locus for Fibromuscular Dysplasia Supporting Its Complex Genetic Pattern of Inheritance
Source: PLoS Genet. 2016 Oct 28;12(10):e1006367. doi: 10.1371/journal.pgen.1006367 (PMC5085032; doi:10.1371/journal.pgen.1006367)
Supplement: S1 Table — SNP, Single Nucleotide polymorphism; Chr, Chromosome; EA, Effect allele; OR, Odds-Ratio; CI, Confidence Intervalle. aFor genic SNPs, the relevant gene is listed; for intergenic SNPs nearest upstream and downstream genes are listed. bSNPs added despite a P value in the discovery stage were above the threshold given their location in or near PHACTR1, the most associated locus. (DOCX) [file pgen.1006367.s004.docx]

**S1 Table. Association of SNPs selected for follow-up in ARCADIA and PPS3 case control analyses.**

| **SNP** | **Locus^a^** | **Chr** | **EA** | **EA Frequency** | **Sample** | **Discovery** | | **Follow-up I** | |
| --- | --- | --- | --- | --- | --- | --- | --- | --- | --- |
|  |  |  |  |  |  | **OR (95% CI)** | **P Value** | **OR (95% CI)** | **P Value** |
| rs6703791 | *IGSF3* | 1 | C | 0.17 | All | 1.55 (1.21-1.99) | 4.98 × 10^−4^ | 0.92 (0.77-1.11) | 0.39 |
|  |  |  |  | 0.16 | Females | 1.72 (1.28-2.30) | 3.29 × 10^−4^ | 0.93 (0.75-1.16) | 0.51 |
|  |  |  |  | 0.17 | Multifocals | 1.65 (1.24-2.20) | 6.23 × 10^−4^ | 0.88 (0.70-1.10) | 0.25 |
| rs1332844^b^ | *PHACTR1* | 6 | C | 0.37 | All | 1.24 (1.00-1.53) | 0.05 | 1.27 (1.10-1.48) | 1.72 × 10^−3^ |
|  |  |  |  | 0.36 | Females | 1.38 (1.07-1.76) | 0.01 | 1.55 (1.29-1.86) | 2.02 × 10^−6^ |
|  |  |  |  | 0.37 | Multifocals | 1.30 (1.01-1.66) | 0.04 | 1.36 (1.13-1.62) | 9.34 × 10^−4^ |
| rs9369640^b^ | *PHACTR1* | 6 | C | 0.37 | All | 1.26 (1.02-1.56) | 0.03 | 1.29 (1.11-1.49) | 8.45 × 10^−4^ |
|  |  |  |  | 0.36 | Females | 1.40 (1.10-1.80) | 7.23 × 10^−3^ | 1.49 (1.25-1.78) | 1.03 × 10^−5^ |
|  |  |  |  | 0.37 | Multifocals | 1.32 (1.03-1.69) | 0.03 | 1.35 (1.14-1.61) | 7.50 × 10^−4^ |
| rs9349379 | *PHACTR1* | 6 | A | 0.59 | All | 1.65 (1.32-2.07) | 1.47 × 10^−5^ | 1.32 (1.12-1.54) | 7.21 × 10^−4^ |
|  |  |  |  | 0.57 | Females | 1.99 (1.51-2.62) | 8.16 × 10^−7^ | 1.46 (1.20-1.77) | 1.23 × 10^−4^ |
|  |  |  |  | 0.59 | Multifocals | 1.75 (1.34-2.29) | 4.41 × 10^−5^ | 1.37 (1.13-1.65) | 1.39 × 10^−3^ |
| rs4357136^b^ | *PHACTR1* | 6 | T | 0.53 | All | 1.26 (1.02-1.55) | 0.03 | 1.04 (0.90-1.21) | 0.58 |
|  |  |  |  | 0.51 | Females | 1.29 (1.01-1.65) | 0.04 | 1.06 (0.88-1.27) | 0.53 |
|  |  |  |  | 0.53 | Multifocals | 1.17 (0.92-1.50) | 0.21 | 1.12 (0.94-1.34) | 0.21 |
| rs436268^b^ | *PHACTR1* | 6 | C | 0.93 | All | 1.37 (0.87-2.16) | 0.18 | 0.92 (0.67-1.26) | 0.61 |
|  |  |  |  | 0.93 | Females | 1.28 (0.76-2.16) | 0.35 | 1.02 (0.69-1.53) | 0.91 |
|  |  |  |  | 0.93 | Multifocals | 1.40 (0.81-2.42) | 0.23 | 0.91 (0.63-1.31) | 0.60 |
| rs2399553 | *LOC101928322 / CELF2* | 10 | T | 0.21 | All | 1.54 (1.22-1.96) | 3.18 × 10^−4^ | 0.86 (0.72-1.02) | 0.08 |
|  |  |  |  | 0.21 | Females | 1.69 (1.27-2.26) | 3.67 × 10^−4^ | 0.84 (0.69-1.04) | 0.11 |
|  |  |  |  | 0.21 | Multifocals | 1.56 (1.19-2.06) | 1.44 × 10^−3^ | 0.83 (0.68-1.03) | 0.09 |
| rs10837607 | *LRRC4C* | 11 | A | 0.12 | All | 1.52 (1.14-2.02) | 3.95 × 10^−3^ | 1.08 (0.87-1.33) | 0.49 |
|  |  |  |  | 0.12 | Females | 1.52 (1.08-2.15) | 0.02 | 1.06 (0.82-1.35) | 0.67 |
|  |  |  |  | 0.12 | Multifocals | 1.76 (1.27-2.43) | 6.29 × 10^−4^ | 1.17 (0.91-1.48) | 0.22 |
| rs1303 | *SERPINA1* | 14 | G | 0.23 | All | 1.57 (1.24-1.98) | 2.13 × 10^−4^ | 1.01 (0.85-1.19) | 0.92 |
|  |  |  |  | 0.25 | Females | 1.39 (1.04-1.86) | 0.03 | 1.00 (0.82-1.23) | 0.97 |
|  |  |  |  | 0.23 | Multifocals | 1.65 (1.25-2.18) | 4.21 × 10^−4^ | 1.02 (0.83-1.25) | 0.85 |
| rs11636613 | *TSPAN3* | 15 | A | 0.27 | All | 1.44 (1.15-1.80) | 1.30 × 10^−3^ | 1.03 (0.88-1.21) | 0.71 |
|  |  |  |  | 0.27 | Females | 1.44 (1.09-1.89) | 9.64 × 10^−3^ | 0.97 (0.80-1.18) | 0.77 |
|  |  |  |  | 0.27 | Multifocals | 1.68 (1.29-2.18) | 1.03 × 10^−4^ | 0.96 (0.79-1.16) | 0.66 |
| rs7188856 | *NAGPA* | 16 | A | 0.30 | All | 1.42 (1.14-1.75) | 1.49 × 10^−3^ | 0.93 (0.80-1.09) | 0.38 |
|  |  |  |  | 0.33 | Females | 1.26 (0.98-1.61) | 0.07 | 0.92 (0.77-1.12) | 0.42 |
|  |  |  |  | 0.30 | Multifocals | 1.54 (1.21-1.98) | 5.88 × 10^−4^ | 0.99 (0.82-1.19) | 0.89 |
| rs944895 | *LAMA5* | 20 | A | 0.61 | All | 1.45 (1.17-1.81) | 8.70 × 10^−4^ | 1.07 (0.92-1.25) | 0.39 |
|  |  |  |  | 0.61 | Females | 1.40 (1.08-1.82) | 0.01 | 1.04 (0.87-1.26) | 0.65 |
|  |  |  |  | 0.61 | Multifocals | 1.30 (1.01-1.68) | 0.04 | 1.13 (0.93-1.36) | 0.21 |
| rs228104 | *SLC37A1* | 21 | G | 0.09 | All | 1.55 (1.12-2.14) | 8.27 × 10^−3^ | 1.02 (0.81-1.28) | 0.88 |
|  |  |  |  | 0.08 | Females | 1.96 (1.32-2.90) | 8.12 × 10^−4^ | 1.05 (0.80-1.38) | 0.73 |
|  |  |  |  | 0.09 | Multifocals | 2.25 (1.59-3.20) | 5.82 × 10^−6^ | 0.98 (0.75-1.30) | 0.91 |
