# Supplementary material for: PHACTR1 Is a Genetic Susceptibility Locus for Fibromuscular Dysplasia Supporting Its Complex Genetic Pattern of Inheritance
Source: PLoS Genet. 2016 Oct 28;12(10):e1006367. doi: 10.1371/journal.pgen.1006367 (PMC5085032; doi:10.1371/journal.pgen.1006367)
Supplement: S2 Table — aAll (N = 2,458), Female (N = 975) and Males only (N = 1,483) participants of the PPS3. βq.t. is the effect size estimated using quantile-transformed values and β is the effect size estimated using untransformed values. P values indicated are for the analyses of quantile transformed values. Age, sex, body surface area (BSA), smoking status and cholesterol were included as covariables in all regression models. MBP was an additional covariable for the following carotid geometry (IMT, Dext, Dint and WLR) and arterial stiffness (Stiffness, Dist, Comp and WCSA) parameters. (DOCX) [file pgen.1006367.s005.docx]

**S2 Table. Association of rs9349379 in *PHACTR1* with carotid artery parameters in PPS3 controls.**

|  |  | **Additive model** | | | **Recessive model** | | |
| --- | --- | --- | --- | --- | --- | --- | --- |
| **Carotid geometry** | **Sample^a^** | **β_q.t._ (95% CI)** | **β** | ***P*** | **β_q.t._ (95% CI)** | **β** | ***P*** |
| Intima–media thickness (IMT), μm | All | 0.070 (0.034 to 0.106) | 11.65 | 1.65 × 10^-4^ | 0.078 (0.042 to 0.115) | 18.97 | 2.44 × 10^-5^ |
|  | Females | 0.095 (0.038 to 0.152) | 14.83 | 0.001 | 0.095 (0.039 to 0.152) | 22.09 | 0.001 |
|  | Males | 0.056 (0.009 to 0.104) | 9.69 | 0.02 | 0.069 (0.022 to 0.116) | 17.12 | 0.004 |
| External diameter (Dext), mm | All | 0.029 (-0.005 to 0.062) | 0.03 | 0.10 | 0.037 (0.004 to 0.071) | 0.05 | 0.03 |
|  | Females | 0.021 (-0.037 to 0.079) | 0.02 | 0.47 | 0.026 (-0.032 to 0.084) | 0.04 | 0.37 |
|  | Males | 0.039 (-0.008 to 0.086) | 0.04 | 0.11 | 0.050 (0.003 to 0.098) | 0.07 | 0.04 |
| Internal diameter (Dint), mm | All | 0.003 (-0.031 to 0.038) | 0.006 | 0.85 | 0.009 (-0.025 to 0.044) | 0.02 | 0.59 |
|  | Females | -0.018 (-0.079 to 0.042) | -0.010 | 0.56 | -0.014 (-0.074 to 0.047) | -0.009 | 0.66 |
|  | Males | 0.020 (-0.029 to 0.069) | 0.02 | 0.43 | 0.026 (-0.023 to 0.075) | 0.03 | 0.29 |
| Wall to lumen ratio (WLR) | All | 0.059 (0.022 to 0.097) | 0.004 | 0.002 | 0.063 (0.026 to 0.101) | 0.006 | 8.24 × 10^-4^ |
|  | Females | 0.093 (0.032 to 0.153) | 0.006 | 0.003 | 0.091 (0.030 to 0.151) | 0.009 | 0.003 |
|  | Males | 0.041 (-0.008 to 0.091) | 0.003 | 0.10 | 0.050 (0.001 to 0.099) | 0.005 | 0.05 |
| Circumferential wall stress at DBP (CWS-DBP), kPa | All | -0.055 (-0.091 to -0.018) | -0.72 | 0.004 | -0.064 (-0.101 to -0.028) | -1.19 | 6.04 × 10^-4^ |
|  | Females | -0.062 (-0.122 to -0.002) | -0.71 | 0.04 | -0.066 (-0.126 to -0.006) | -1.02 | 0.03 |
|  | Males | -0.052 (-0.102 to -0.003) | -0.72 | 0.04 | -0.066 (-0.115 to -0.017) | -1.29 | 0.009 |
| **Arterial Stiffness** |  |  |  |  |  |  |  |
| Stiffness, m × s^-1^ | All | -0.027 (-0.065 to 0.011) | 0.05 | 0.16 | -0.025 (-0.063 to 0.014) | 0.07 | 0.21 |
|  | Females | -0.008 (-0.068 to 0.051) | 0.01 | 0.79 | -0.021 (-0.080 to 0.039) | 0.07 | 0.49 |
|  | Males | -0.044 (-0.093 to 0.006) | 0.07 | 0.09 | -0.027 (-0.076 to 0.023) | 0.06 | 0.29 |
| Cross-sectional distensibility, (Dist), kPa^-1^ × 10^-3^ | All | -0.029 (-0.067 to 0.009) | -0.33 | 0.13 | -0.028 (-0.065 to 0.010) | -0.40 | 0.15 |
|  | Females | -0.007 (-0.067 to 0.052) | -0.10 | 0.81 | -0.016 (-0.075 to 0.043) | -0.21 | 0.59 |
|  | Males | -0.047 (-0.096 to 0.002) | -0.51 | 0.06 | -0.036 (-0.085 to 0.014) | -0.51 | 0.16 |
| Cross-sectional compliance, (Comp), mm^2^ × kPa^-1^ × 10^-3^ | All | -0.025 (-0.063 to 0.013) | -0.07 | 0.19 | -0.022 (-0.060 to 0.015) | -0.08 | 0.24 |
|  | Females | -0.021 (-0.082 to 0.040) | -0.06 | 0.50 | -0.034 (-0.094 to 0.027) | -0.13 | 0.28 |
|  | Males | -0.030 (-0.081 to 0.020) | -0.08 | 0.24 | -0.016 (-0.067 to 0.034) | -0.04 | 0.53 |
| Young's elastic modulus, (Ceinc), kPa | All | 0.011 (-0.028 to 0.051) | 2.95 | 0.57 | 0.007 (-0.033 to 0.046) | 4.04 | 0.74 |
|  | Females | -0.010 (-0.072 to 0.052) | -4.07 | 0.75 | 0.004 (-0.058 to 0.066) | 4.38 | 0.90 |
|  | Males | 0.029 (-0.022 to 0.080) | 8.25 | 0.26 | 0.009 (-0.042 to 0.060) | 3.88 | 0.74 |
| Wall cross-sectional area (WCSA), mm2 | All | 0.060 (0.025 to 0.095) | 0.24 | 8.67 × 10^-4^ | 0.070 (0.035 to 0.105) | 0.41 | 9.83 × 10^-5^ |
|  | Females | 0.078 (0.022 to 0.133) | 0.30 | 0.006 | 0.080 (0.025 to 0.135) | 0.46 | 0.005 |
|  | Males | 0.051 (0.005 to 0.098) | 0.21 | 0.03 | 0.065 (0.019 to 0.112) | 0.39 | 0.006 |
| **Blood pressure** |  |  |  |  |  |  |  |
| Systolic blood pressure (SBP), mm Hg | All | 0.052 (0.013 to 0.090) | 0.70 | 0.009 | 0.041 (0.002 to 0.079) | 0.76 | 0.04 |
|  | Females | 0.066 (0.004 to 0.129) | 0.90 | 0.04 | 0.055 (-0.008 to 0.117) | 1.13 | 0.09 |
|  | Males | 0.044 (-0.007 to 0.094) | 0.47 | 0.09 | 0.032 (-0.018 to 0.083) | 0.50 | 0.21 |
| Diastolic blood pressure (DBP), mm Hg | All | 0.001 (-0.038 to 0.040) | 0.0060 | 0.96 | -0.014 (-0.053 to 0.025) | -0.20 | 0.48 |
|  | Females | 0.037 (-0.026 to 0.101) | 0.30 | 0.25 | 0.023 (-0.040 to 0.086) | 0.30 | 0.47 |
|  | Males | -0.022 (-0.073 to 0.029) | -0.20 | 0.39 | -0.038 (-0.088 to 0.013) | -0.52 | 0.15 |
| Mean blood pressure (MBP), mm Hg | All | 0.021 (-0.018 to 0.060) | 0.21 | 0.29 | 0.008 (-0.031 to 0.046) | 0.10 | 0.70 |
|  | Females | 0.048 (-0.014 to 0.111) | 0.51 | 0.13 | 0.038 (-0.025 to 0.100) | 0.57 | 0.24 |
|  | Males | 0.003 (-0.048 to 0.054) | 0.02 | 0.90 | -0.013 (-0.064 to 0.038) | -0.19 | 0.62 |
| Centrol pulse pressure, (CPP), mm Hg | All | 0.061 (0.023 to 0.099) | 0.62 | 0.002 | 0.065 (0.027 to 0.103) | 0.62 | 8.57 × 10^-4^ |
|  | Females | 0.036 (-0.024 to 0.096) | 0.39 | 0.24 | 0.043 (-0.017 to 0.103) | 0.39 | 0.16 |
|  | Males | 0.082 (0.033 to 0.132) | 0.79 | 0.001 | 0.081 (0.032 to 0.131) | 0.79 | 0.001 |
